# Ingestion of miso regulates immunological robustness in mice

**DOI:** 10.1371/journal.pone.0261680

**Published:** 2022-01-21

**Authors:** Kunihiko Kotake, Toshihiko Kumazawa, Kiminori Nakamura, Yu Shimizu, Tokiyoshi Ayabe, Takahiro Adachi

**Affiliations:** 1 Ichibiki Co., Ltd., Nagoya, Japan; 2 Department of Precision Health, Medical Research Institute, Tokyo Medical and Dental University, Tokyo, Japan; 3 Faculty of Advanced Life Science, Department of Cell Biological Science, Hokkaido University Graduate School of Life Science, Sapporo, Japan; Northwestern University Feinberg School of Medicine, UNITED STATES

## Abstract

In Japan, there is a long history of consumption of miso, a fermented soybean paste, which possesses beneficial effects on human health. However, the mechanism behind these effects is not fully understood. To clarify the effects of miso on immune cells, we evaluated its immunomodulatory activity in mice. Miso did not alter the percentage of B and T cells in the spleen; however, it increased CD69^+^ B cells, germinal center B cells and regulatory T cells. Anti-DNA immunoglobulin M antibodies, which prevent autoimmune disease, were increased following ingestion of miso. Transcriptome analysis of mouse spleen cells cultured with miso and its raw material revealed that the expression of genes, including interleukin (IL)-10, IL-22 and CD86, was upregulated. Furthermore, intravital imaging of the small intestinal epithelium using a calcium biosensor mouse line indicated that miso induced Ca^2+^ signaling in a manner similar to that of probiotics. Thus, ingestion of miso strengthened the immune response and tolerance in mice. These results appear to account, at least in part, to the salubrious effects of miso.

## Introduction

Miso is a traditional fermented food consumed in Japan. It is classified according to the type of mold (koji), fermented by *Aspergillus* or other microorganisms. Soybean-miso (mame-miso) is prepared by mixing fermented beans and salt. It primarily consists of soybeans and is rich in proteins (including amino acids and peptides); thus, it is characterized by a strong umami and a dark color.

There are various health benefits associated with eating miso. Daily consumption of miso soup may improve skin moisture, and some ingredients in miso stimulate the increase of ceramides in the stratum corneum [1]. Miso also has an anti-hypertensive effect [2,3] and is known to act on the brain to suppress salt-sensitive sympathoexcitation [4,5]. Moreover, it has been reported that miso has anti-cancer activity against breast [6], gastric [7] and colon cancers [8]. The consumption of miso has also been reported to exert health effects, such as fat suppression [9], anti-inflammation [10] and stroke prevention [11].

Miso contains various microorganisms, such as *Aspergillus*, yeast and lactic acid bacteria (LAB). In recent years, these microorganisms have attracted attention as a source of probiotics. *Aspergillus oryzae* has been shown to alter the intestinal bacterial flora through the production of glycosylceramide [12]. There are many types of LABs, and several strains have been reported to show beneficial health effects, such as activating immunoglobulin (Ig) A production [13,14] and alleviating allergy symptoms [15]. Recently, we have identified miso-derived LABs that promote interleukin (IL)-22 production in B cells and improve skin barrier [13,16].

Therefore, miso is expected to have many beneficial effects on human health, but it is not clear how immune cells are regulated by miso intake. In this study, we evaluated immune cells in mice fed a diet containing miso.

## Materials and methods

### Ethics statement

All mice were maintained at our animal facility under specific-pathogen-free (SPF) conditions in accordance with the animal care guidelines of Tokyo Medical and Dental University. All animal experiments were approved by the Committee for Animal Care at Tokyo Medical and Dental University (approval number A2019-207C4).

### Miso and raw materials

We used the following food materials: soybean-miso (Ichibiki), rice-miso (Ichibiki), white hilum soybean cultivar Tanrei made in Japan (Kushida), black hilum soybean made in the USA (Kanematsu), white hilum soybean made in the USA (Kanematsu), rice cultivar Calrose made in the USA (Kantokokuhun), rice made in Japan (Kimura-Shoten) and rice cultivar Koshihikari (YAMATO SANGYO). Soybeans and rice were crushed and used as a powder.

### Cells and mice

C57BL/6 mice were fed a diet supplemented with 5% miso (soybean-miso) under SPF conditions. The ingredients of the miso are provided in Table 1. Miso mixed feed was prepared by combining with CLEA CE-2. As a control, feed was prepared by adding salt to CLEA CE-2. These feeds were standardized at a salt content of 1.4 w/w%. IL-22 deficient mice were generated by a genome editing system of the CRISPR/Cas9 as described previously [17]. A transgenic mouse line expressing YC3.60 for intravital imaging was described previously [18]. After euthanasia due to cervical vertebral dislocation, spleens were removed from mice and spleen cells were prepared as described previously [19].

**Table 1:** Ingredient of miso per 100 g.

Ingredient	Unit	Soybean-miso	Rice-miso
**Energy**	**Kcal**	216	195
**Protein**	**G**	18.0	11.0
**Fat**	**G**	9.2	6.5
**Carbohydrate**	**G**	14.5	23.1
**Moisture**	**G**	45.0	46.2
**Ash**	**G**	12.4	13.2
**Salt equivalent**	**G**	4.1	4.8

### Flow cytometry

The cells were analyzed on a MACSQuant Flow Cytometer (MiltenyiBiotec) using the following specific antibodies: VioletFluor^TM^ 450-conjugated anti-B220 (TONBO Biosciences), Brilliant Violet 510^TM^-conjugated anti-CD4 (BioLegend), Brilliant Violet 510^TM^-conjugated anti-CD8a (BioLegend), phycoerythrin (PE)-conjugated anti-CD138 (BioLegend), Alexa Fluor 647-conjugated anti-GL7 (BioLegend), PE-conjugated anti-CD62L (BD Biosciences), allophycocyanin (APC)-conjugated anti-CD44 (eBioscience), APC-conjugated anti-CD86 (TONBO Biosciences), PE-conjugated anti-CD69 (BioLegend), PE-conjugated anti-CD23 (BioLegend), Alexa Fluor 647-conjugated anti-CD21 (BD Pharmingen), PE-conjugated anti-PD-1 (TONBO Biosciences), Brilliant Violet 421^TM^-conjugated anti-CXCR5 (BioLegend), Alexa Fluor 647-conjugated ICOS (BioLegend), eFour450-conjugated anti-CD127 (eBioscience), PE-conjugated anti-CD25 (TONBO Biosciences) and APC-conjugated anti-CTLA4 (TONBO Biosciences). Data analysis was conducted with FlowJo software (v7.6.5; FLOWJO, LLC).

### DNA extraction

Two-hundred mg of fecal samples was subjected to total DNA extraction using QIAamp Fast DNA Stool Mini Kit (QIAGEN) following the manufacturer’s instruction. Final DNA concentrations were estimated based on 260 nm absorbance using a Nanodrop 2000 spectrometer (Thermo Fischer Scientific).

### Preparation of 16S rRNA gene amplicon sequence library

V3-V4 variable region of 16S rDNA in each fecal DNA sample was amplified by polymerase chain reaction (PCR) using universal primer set of Bakt 341F (5-cctacgggnggcwgcag) and Bakt 805R (5-gactachvgggtatctaatcc) [20]. 1^st^ PCR was conducted in 25 μL of reaction mixtures containing 12.5 ng of fecal DNA, 200 nM of each primer, and 1x KAPA HiFi Hot Start Ready Mix (Kapa Biosystems) under the following parameters: 95°C for 3 min, 25 cycles of 95°C for 30 sec, 55°C for 30 sec and 72°C for 30 sec, followed by 72°C for 5 min. 1^st^ PCR products were purified by AMPure XP beads (Beckman Coulter) and subjected to 2^nd^ PCR for adding sequencing adapters containing sample specific 8 bp barcodes to the 3’- and 5’- ends by using the Nextera XT Index Kit v2 Set B (Illumina). 2^nd^ PCR was conducted in 50 μL of reaction mixtures containing 5 μL of 1st PCR products, 5 μL of each indexing primer and 1x KAPA HiFi Hot Start Ready Mix under the following conditions: 95°C for 3 min, 8 cycles of 95°C for 30 sec, 55°C for 30 sec and 72°C for 30 sec, followed by 72°C for 5 min. Each 2^nd^ PCR amplicon was purified, quantified by the Qubit dsDNA HS Assay Kit (Thermo Fischer Scientific), and adjusted to 4 nM. Then, 4μL of each amplicon were pooled and quantified by KAPA Library Quantification Kit Lightcycler 480 qPCR Mix (Kapa Biosystems) and adjusted to 4 pM. The amplicon library was mixed with 5% of equimolar PhiX Control v3 (Illumina) and sequenced on a MiSeq instrument using the MiSeq 600-cycle v3 kit (Illumina) by pair-end sequencing mode.

### Metagenomic analysis

Pair-end sequencing reads generated from Miseq were demultiplexed and imported into QIIME2 pipeline (v2019.7) [21]. Quality-filtering, denoising, and removal of chimeric sequences were conducted by DADA2 plugin [22] with following parameters;—p-trim-left-f 17,—p-trim-left-r 21,—p-trunc-len-f 280,—p-trunc-len-r 200,—p-max-ee-f 2 and—p-max-ee-r 2. Phylogenic tree was generated by FastTree [23] after alignment with MAFFT [24]. Taxonomy of each feature were assigned using a naïve-bayes classifier trained on 16S rRNA gene OTUs clustered at 99% similarities within the Silva database (v132). Estimation of α-diversity (Simpson index) and β-diversity (unweighted UniFrac distance) was conducted by QIIME2 workflow. Statistical significance of α-diversity was tested by Permutational multivariant analysis of variance (PERMANOVA) test in QIIME2 pipeline.

### Measurement of serum Ig levels

Serum Ig levels were measured as described previously [25] using enzyme-linked immunosorbent assays (ELISAs). Sonicated herring sperm DNA (10 μg/ml, Sigma-Aldrich) was coated onto ELISA plates. Alkaline phosphatase-conjugated anti-IgM and anti-IgG (Southern Biotech) antibodies (Abs) were used.

### Statistics

Experimental data are indicated as the mean ± standard deviation (S.D.). Statistical significance was evaluated using a two-tailed Student’s t-test for unpaired data. P-values < 0.05 were considered statistically significant.

### Transcriptome analyses

A total of 4 × 10^6^ spleen cells were cultured in 1 mL RPMI 1640 medium containing 10% FCS with 10 μg of miso or miso ingredients powders for 2 d. Total RNA was prepared from spleen cells using ISOGEN II (Nippon Gene) and subjected to RNA sequencing analysis (GENEWIZ). RNA-Seq library construction, next generation sequencing, and bioinformatics analyses were done using GENEWIZ. Briefly, total RNA was quantified and qualified using the Qubit RNA Assay (Invitrogen) and RNA ScreenTape (TapeStation; Agilent Technologies). Poly(A) mRNA was enriched using magnetic bead-conjugated oligo(dT), and library preparation for high-throughput sequencing was conducted according to the manufacturer’s instructions (NEBNext Ultra II RNA Library Prep Kit for Illumina; New England BioLabs). Approximately 250 ng of total RNA was used for mRNA selection, and adapter-ligated double stranded cDNA fragment was amplified using 12 cycles of PCR, which incorporated Illumina P5/P7 adapters and a sample-specific barcode sequence. Fragment size and quantity of the libraries were confirmed using Qubit DNA Assay (Invitrogen) and DNA ScreenTape (TapeStation; Agilent Technologies). Libraries with unique sample barcodes were pooled together and loaded onto an Illumina HiSeq/NovaSeq instrument according to the manufacturer’s instructions (Illumina). Sequencing was carried out using a 150-bp paired-end configuration. Image analysis, base calling, and demultiplex were performed using the Illumina standard software. Approximately 20 M paired-end reads (6-Gb output in 150-bp paired-end configuration) per sample were obtained. The raw sequencing reads were filtered to remove adapter and low-quality reads. The resulting clean reads were used for mapping against a reference genome (*Mus musculus*; Ensembl/GRCm38), quantifying gene expression, identifying differential gene expression, and other downstream analyses. Gene expression ratio over 1.5 indicate significant differences.

### Intravital microscopy

Intestinal epithelial cells (IECs) from anesthetized mice were imaged. Mice were anesthetized by intraperitoneal administration of 50 mg/kg pentobarbital, and the small intestinal tract was surgically opened lengthwise, placed on a slide glass with a pump attached, and immobilized on a microscope stage. For image acquisition, a Nikon A1 laser-scanning confocal microscope with a 20× objective and NIS-Elements AR software were used as previously described [25]. We used a dichroic mirror (DM457/514) and two bandpass emission filters (482/35 for cyan fluorescent protein [CFP] and 540/30 for yellow fluorescent protein [YFP]). The YFP/CFP ratio was obtained by excitation at 458 nm. Acquired images were analyzed using NIS-Elements software (Nikon).

## Results

### Miso acts on immune cells as an activator and suppressor

**Fig 1:**
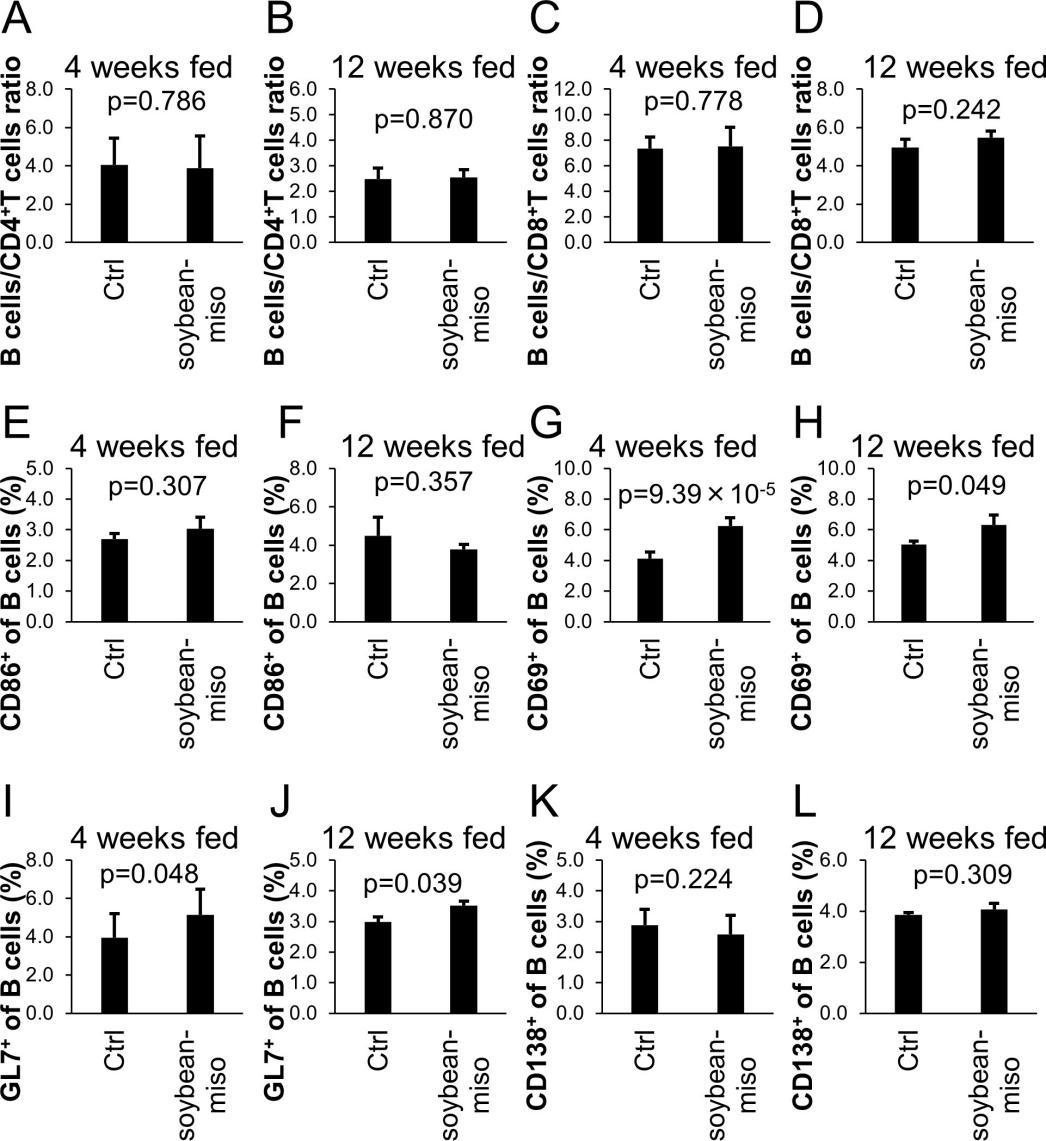
The effect of miso on B cells and T cells in mouse spleens. (A) The ratio of B220^+^/CD4^+^ cells fed for 4 weeks. (B) The ratio of B220^+^/CD4^+^ cellsfed for 12 weeks. (C) The ratio of B220^+^/CD8a^+^ cells fed for 4 weeks. (D) The ratio of B220^+^/CD8a^+^ cells fed for 12 weeks. (E) The rate of CD86^+^/B cells fed for 4 weeks. (F) The rate of CD86^+^/B cells fed for 12 weeks. (G) The rate of CD69^+^/B cells fed for 4 weeks. (H) The rate of CD69^+^/B cells fed for 12 weeks. (I) The rate of GL7^+^/B cells fed for 4 weeks. (J) The rate of GL7^+^/B cells fed for 12 weeks. (K) The rate of CD138^+^/B cells fed for 4 weeks. (L) The rate of CD138^+^/B cells fed for 12 weeks. Diet containing 5% soybean-miso was fed to C57BL/6 mice for 4 (n = 12) or 12 (n = 3) weeks. Then, spleen samples were harvested and analyzed using flow cytometry. Bars indicate mean ± S.D. P-values relative to the control using a t-test.

Since miso and miso-derived microorganisms have been shown to modulate the immune response [13,16], we examined the effect of miso on B cells and T cells in mice. Mice were fed a diet containing 5% miso for 4 or 12 weeks, and the ratio of B cells and T cells in the spleen were analyzed, specifically, B cells (B220^+^), CD4^+^ and CD8^+^ T cells (S1A and S1B Fig). Regardless of the duration of feeding, the ratio between B cells and T cells in the spleen of miso-treated mice did not differ from those of the control mice (Fig 1A–1D).

Next, we analyzed subsets of B cells. There are several subsets of B cells in the spleen: marginal zone B cells (MZ B cells: CD19^+^CD21^+^CD23^−^), follicular B cells (Fo B cells: CD19^+^CD21^+^CD23^+^) and transitional type 1 B cells (T1 B cells: CD19^+^CD21^-^CD23^-^). We examined subsets of these B cells and found no changes associated with miso intake (S2 Fig).

B cells are activated and differentiate into plasma cells through germinal center (GC) B cells or directly. We examined the activation markers, CD86 and CD69, on spleen B cells (S1C and S1D Fig). Although the populations of CD86^+^ B cells were unaltered, CD69^+^ B cells were increased (Fig 1E–1H). Since activated B cells were increased, we next examined the status of GC B cells (B220^+^GL7^+^) and plasma cells (B220^-^CD138^+^) (S1E and S1F Fig). Ingestion of miso increased the population of GC B cells in the spleen, whereas it did not alter the population of plasma cells (Fig 1I–1L).

**Fig 2:**
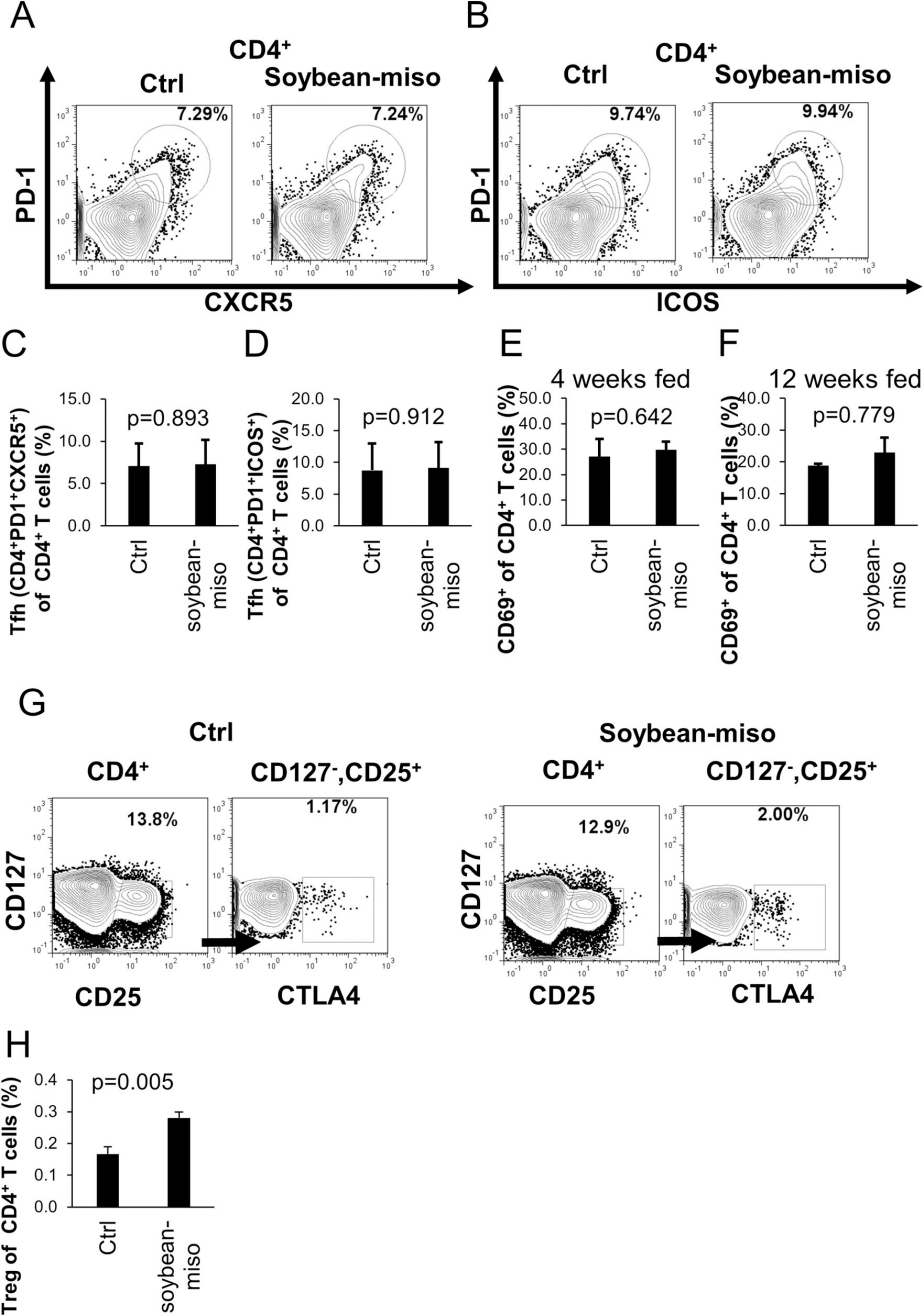
The effect of miso on subsets of T cells in mouse spleens. (A) Examples of the measurement of Tfh (CD4^+^PD-1^+^CXCR5^+^) by flow cytometry are shown. (B) Examples of the measurement of Tfh (CD4^+^PD-1^+^ICOS^+^) by flow cytometry are shown. (C) The rate of Tfh (CD4^+^PD-1^+^CXCR5^+^)/CD4^+^ T cells in spleens of mice fed for 4 weeks. (D) The rate of Tfh (CD4^+^PD-1^+^ICOS^+^)/CD4^+^ T cells in spleens of mice fed for 4 weeks. (E) The rate of CD69^+^/CD4^+^ T cells in mice fed for 4 weeks. (F) The rate of CD69^+^/CD4^+^ T cells in mice fed for 12 weeks. (G) Examples of the measurement of Treg cells by flow cytometry are shown. (H) The rate of Treg/CD4^+^ T cells in mice fed for 4 weeks. Diet containing 5% soybean-miso was fed to C57BL/6 mice for 4 (n = 12) or 12 (n = 3) weeks. Spleen samples were collected and analyzed using flow cytometry. Bars indicate mean ± S.D. P-values relative to the control using a t-test.

We also analyzed subsets of T cells. First, we measured the number of memory T cells (CD4^+^CD44^high^CD62L^−^) and naive T cells (CD4^+^CD44^low^CD62L^+^) in the spleen, however, the population of these cell types was not altered (S3 Fig). Next, we analyzed follicular helper T (Tfh) cells crucial for B cell activation. The Tfh (CD4^+^PD-1^+^CXCR5^+^ or CD4^+^PD-1^+^ICOS^+^) cell population was not altered (Fig 2A–2D). Furthermore, the population of activated CD4 T cells (CD69^+^CD4^+^ T cells) was not altered (Figs 2E, 2F and S1G). In addition, we analyzed regulatory T (Treg) cells crucial for immune tolerance. Splenic Treg (CD4^+^CD127^−^CD25^+^CTLA4^+^) cells were increased in miso-fed mice for 4 weeks compared with that of the control mice (Fig 2G and 2H). Overall, these results show that miso increased both immune-stimulatory and -regulatory cells in mice under steady-state conditions.

Treg cells increased in miso-fed mice. Butyrate which is a metabolite derived from microbiota is known to induce Treg cells [26]. Furthermore, Treg cells induction is known to mediated by food antigen and gut microbiota [27]. To clarify whether increase of Treg cells is mediated by microbiota metabolite, butyrate, we examined fecal microbiota of miso-fed mice. So far, the effect of miso on gut microbiota is limited. Therefore, we used IL-22 deficient mice which is sensitive to diet due to its weak gut barrier function [28]. Fecal microbiota composition of miso-fed mice was almost no differences in α-diversity and β-diversity from that of control diet-fed mice (S4 Fig). Furthermore, relative abundance of Clostridiales including most of butyrate-producing bacteria was almost the same between the two groups. These results suggested that effect of metabolite on miso-mediated immune regulation seems to be marginal.

### Long-term ingestion of miso increases anti-DNA IgM Abs

**Fig 3:**
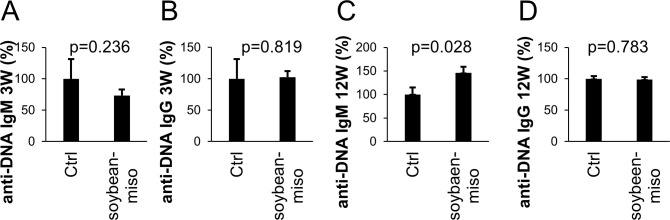
The effect of miso on Anti-DNA IgM and IgG production in serum. (A) Anti-DNA IgM levels in serum from mice fed for 3 weeks. (B) Anti-DNA IgG level in serum from mice fed for 3 weeks. (C) Anti-DNA IgM level in serum of mice fed for 12 weeks. (D) Anti-DNA IgG level in serum of mice fed for 12 weeks. Diet containing 5% soybean-miso was fed to C57BL/6 mice for 3 (n = 3) or 12 (n = 3) weeks. Then, spleen samples were collected and analyzed using flow cytometry. Bars indicate mean ± S.D. P-values relative to the control using a t-test.

Serum IgM is crucial for preventing autoimmunity [29]. Abs of the IgM class prevent further immune response and production of IgG. Autoantibodies of the IgG class specific for DNA have been shown to be pathogenic and trigger autoimmune symptoms, such as lupus nephritis [30]. To confirm the effect of miso on immune tolerance, we measured anti-DNA Abs in the serum. Anti-DNA IgM Abs and anti-DNA IgG Abs in the serum were detected in normal mice and were unchanged in mice fed miso for 3 weeks (Fig 3A and 3B). In mice fed miso for 12 weeks, anti-DNA IgM Abs in the serum were increased, whereas anti-DNA IgG Abs were not altered (Fig 3C and 3D). Thus, protective IgM Abs against autoimmunity were increased upon continued ingestion of miso, suggesting that miso augments beneficial immune reactions for self-tolerance after long-term ingestion of miso.

### Miso directly regulates the expression of genes involved in the immunity of spleen cells in mice

We previously found that *Tetragenococcus halophilus* No.1, which was isolated from miso, regulates gene expression in spleen cells *in vitro* [13]. To confirm whether the miso itself or miso ingredients have the same effect, we performed a transcriptome analysis of spleen cells cultured with these extracts (Tables 2 and 3 and S5 Fig). Similar to *T*. *halophilus* No.1 isolated from miso [13], the expression of CD86, IL-10, IL-22 and Interferon gamma (IFN-γ) were upregulated by miso. In addition, the expression of CD69, which is an activation marker for B cells and T cells, and IL-1β, which is an inflammatory cytokine, were all upregulated by miso. In contrast, soybeans and rice, which are the raw materials of miso, did not show significant upregulation of these genes. These results indicate that miso, but not soybeans and rice, directly regulates the expression of genes involved in the immunity of spleen cells in mice.

**Table 2:** Soybean and rice information.

	Soy-1	Soy-2	Soy-3	Rice-1	Rice-2	Rice-3
**Country of Origin**	Japan	USA	USA	USA	Japan	Japan
**Variety**	Tanrei	Non-classified	Non-classified	Calrose	Non-classified	Koshihikari
**Hilum Color**	White	Black	White	-	-	-

**Table 3:** Gene expression ratio (sample/control).

	Soy Miso	Rice miso	Soy-1	Soy-2	Soy-3	Rice-1	Rice-2	Rice-3
**IL-10**	1.47	**1.51**	1.22	0.97	1.47	1.28	1.28	1.39
**IL-22**	**2.02**	**1.73**	**1.79**	1.04	1.47	1.08	1.05	**1.82**
**IFN-γ**	**2.31**	**2.80**	1.04	1.25	**1.56**	1.42	**1.74**	0.99
**IL-1β**	**1.98**	**1.81**	1.46	1.14	**1.94**	1.10	1.31	**1.51**
**CD86**	1.18	1.36	1.05	1.17	1.15	1.01	1.10	1.25
**CD69**	1.36	**1.51**	1.15	1.17	1.27	1.30	**1.55**	**1.54**

### Effect of miso on Ca^2+^ signaling in the IECs of mice with ubiquitous YC3.60 expression *in vivo*

As mentioned above, the ingestion of miso affects the immune system. The IECs also secrete cytokines and their receptors [31]. For instance, the IECs secrete IL-10 in response to toll-like receptor 4 stimulation [32]. Furthermore, recently the enteric epithelial tuft cells produce IL-25 which is crucial for protection against parasite infection [33]. Thus, the IECs communicating with immune cells play crucial roles in the immune system. Therefore, we examined whether miso stimulates directly the enteric epithelial cells or not. To clarify this, we performed intravital imaging to determine whether miso directly stimulates IECs. We previously established an intravital imaging system for Ca^2+^ signaling in IECs and evaluated the effect of probiotics, including *Lactococcus lactis* and *Bacillus subtilis* [18]. To improve the imaging system, we created a special device that can inject a stimulus solution along with intravital imaging. As shown in S5 Fig, this device together with a cover glass is used to hold the target tissue by compression with a pump. Meanwhile, the solution can reach the tissue through a tube from an injection syringe. This enables stable observation of moving tissues such as that of the intestine.

**Fig 4:**
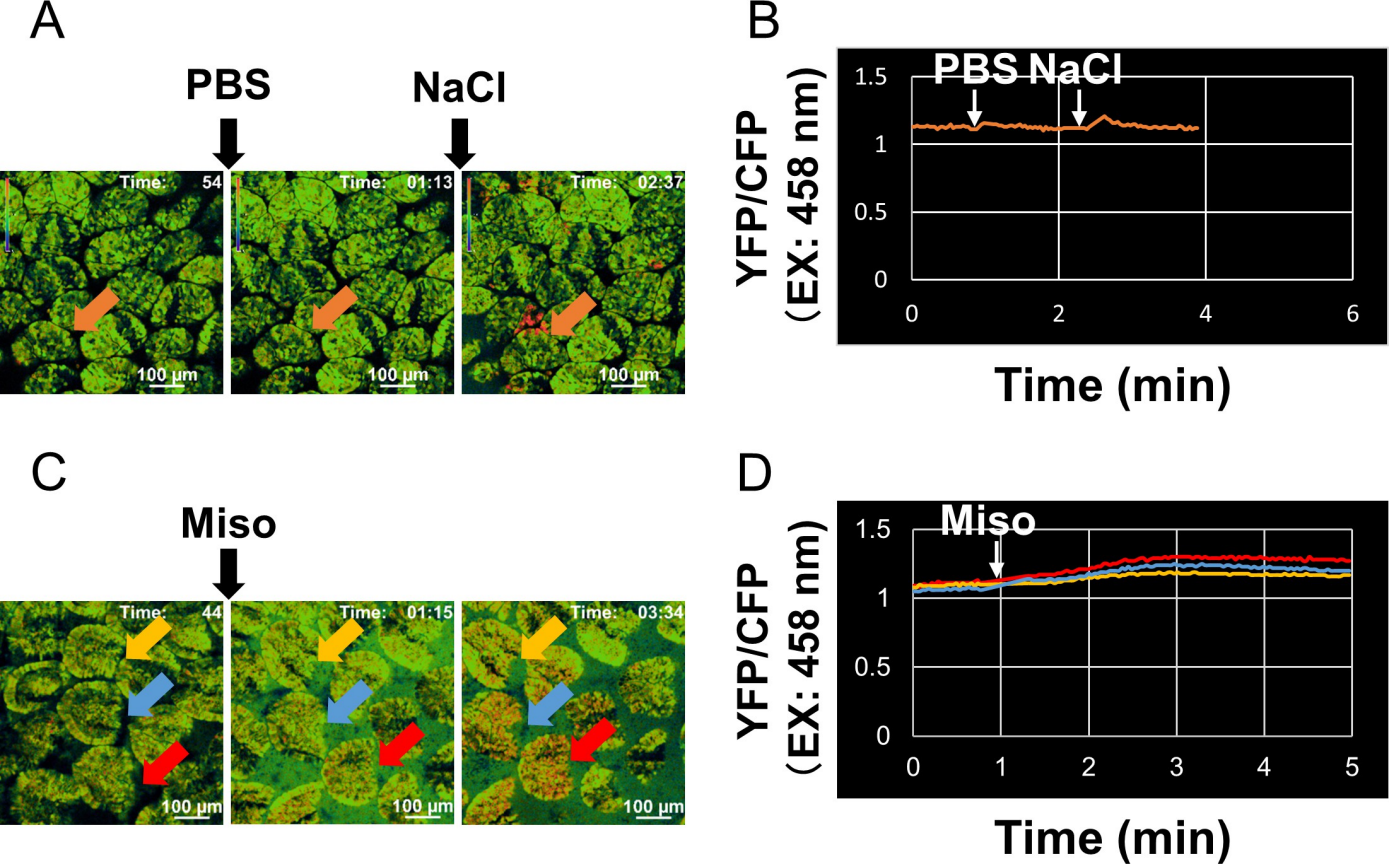
Images of intravital Ca^2+^ signaling in the IECs of a mouse expressing YC3.60. (A) Representative Ca^2+^ signaling of PBS and 1% NaCl images in the intestinal tract of a mouse with ubiquitous YC3.60 expression and SPF conditions. Ratiometric images (YFP/CFP at excitation of 458 nm) are shown. A total of 0.1 ml of PBS or 1% NaCl were added at the indicated time points. (B) Time course of fluorescence intensities of YFP/CFP upon excitation at 458 nm when the intestinal tract was stimulated with PBS and 1% NaCl. (C) Representative Ca^2+^ signaling of PBS and 10% soybean-miso images in the intestinal tract of a mouse with ubiquitous YC3.60 expression and SPF conditions. Ratiometric images (YFP/CFP at excitation of 458 nm) are shown. A total of 0.1 ml of 10% miso in PBS was added at the indicated time point. (D) Time course for fluorescence intensities of YFP/CFP upon excitation at 458 nm when the intestinal tract was stimulated with miso and measured in randomly selected regions (n = 3). Scale bar, 100 μm; frames = 131. Spontaneous Ca^2+^ signals are indicated by arrows.

Using an improved imaging system, we tested whether miso induces Ca^2+^ signaling in IECs. The miso extract was prepared by diluting 10 times with water and then filtered. As a control, we adjusted the NaCl concentration to 1% with water. We surgically opened the small intestinal tract of the mice exhibiting ubiquitous YC3.60 expression [18], placed it on the stage of the confocal microscope, and added PBS, 1% NaCl and miso extract to the intestinal epithelium (S6 Fig). Adding 1% NaCl temporarily increased intracellular Ca^2+^ concentration, whereas the addition of PBS did not affect intracellular Ca^2+^ concentration in the IECs (Fig 4A and 4B and S1 Video). However, adding miso strikingly increased intracellular Ca^2+^ concentration in the IECs (Fig 4C and 4D and S2 Video). Miso induced a gradual elevation of intracellular Ca^2+^ concentration, which is similar to that of probiotics such as *L*. *lactis* and *B*. *subtilis* [18]. Thus, miso directly affected IECs to a similar extent as probiotics.

## Discussion

In this study, we found that the ingestion of miso increased the expression of CD69 on B cells and GC B cells, suggesting that miso enhances part of the process by which B cells differentiate into plasma cells. Furthermore, consuming miso increased the amount of Treg cells and increased anti-DNA IgM Abs levels. Miso upregulated the expression of IL-10, IL-22 and CD86 mRNA. Miso evoked Ca^2+^ signaling in the IEC similar to probiotics. These results suggest that miso regulates immunity by affecting immune activation and immune tolerance.

As part of the daily diet in Japan, miso has traditionally been consumed and suggested to be beneficial to human health. Indeed, it was shown that the consumption of a large amount of soy products in the Asian population can effectively control breast cancer, but this effect has not been observed in Westerners who consume a small amount of soy products [34]. Soybean protein has also been reported to inhibit blood pressure [35] and exhibit antioxidant activity [36], whereas soybean isoflavones exhibit anti-cancer effects [37,38] and promote bone formation [39]. Furthermore, intake of miso soup before pregnancy significantly reduces the risk of early preterm birth [40]. In this study, we showed that miso augmented both immunity and immune tolerance under steady-state conditions as activated B cells and Treg cells were increased upon ingestion of miso. These results suggest that miso strengthens immune robustness and prevents both autoimmunity and infectious diseases, resulting in the maintenance of good health.

LAB isolated from miso has been reported to suppress allergies [41,42], and other studies have reported that it enhances immunity, in part, by activating B cells [13]. In this study, we found that miso regulates immunity in the same way as LAB isolated from miso. Furthermore, we conducted a transcriptome analysis of miso and its raw materials, which revealed that miso regulates the expression of genes involved in immunity. This result is similar to that observed with *T*. *halophilus* No.1, which we previously isolated from miso rather than the soybeans and rice that are the raw materials for miso. From these results, we conclude that the LAB in miso contributes, in part, to the health effects of miso.

Intravital Ca^2+^ imaging of the small intestinal epithelium showed that miso stimulates IECs. The kinetics of Ca^2+^ signaling mediated by miso resembles that of probiotics [18]. Based on this similarity, it is likely that probiotics contained in miso may induce Ca^2+^ signaling. Miso seems to affect the immune system by directly stimulating immune cells and epithelial cells. In the small intestine, there is an abundance of endocrine cells, immune cells and nerve cells (neurons and glial cells), which functionally interact with one another [43]. Besides microbiotics, miso contains abundant amino acids. Some amino acids such as glutamate have been shown to induce the production of hormones in enteroendocrine cells [44,45]. It is also possible that these components contribute to Ca^2+^ signaling via enteroendocrine cells, although further analyses of this process are needed.

Collectively, we showed the effect of miso on immunological robustness in mice. This may contribute to Japanese longevity and health. Our study may help to clarify the beneficial effects of miso on human health in the near future.

## Supporting information

S1 Fig Examples of mice spleen cells measurement by flow cytometry. (A) B220 and CD4. (B) B220 and CD8a. (C) B220 and CD86. (D) B220 and CD69. (E) B220 and GL7. (F) B220 and CD138. (G) CD4 and CD69.
(TIF)

S2 Fig The effect of miso on subsets of B cells in mouse spleen. (A) Examples of the measurement by flow cytometryare shown. (B) The rate of CD21^+^CD23^−^/B cells in mice fed for 4 weeks. (C) The rate of CD21^+^CD23^−^/B cells in mice fed for 12 weeks. (D) The rate of CD21^+^CD23^+^/B cells in mice fed for 4 weeks. (E) The rate of CD21^+^CD23^+^/B cells in mice fed for 12 weeks. (F) The rate of CD21^-^CD23^-^/B cells in mice fed for 4 weeks. (G) The rate of CD21^-^CD23^-^/B cells in mice fed for 12 weeks. Diet containing 5% soybean-miso was fed to C57BL/6 mice for 4 (n = 12) or 12 (n = 3) weeks. Spleen samples were collected and were analyzed using flow cytometry. Bars indicate mean ± S.D. P-values relative to the control using a t-test.
(TIF)

S3 Fig The effect of miso on memory and naive T cells in spleen. (A) Examples of the measurement by flow cytometry. (B) The rate of CD44^high^CD62L^−^/CD4^+^ T cells in mice fed for 4 weeks. (C) The rate of CD44^high^CD62L^−^/CD4^+^ T cells in mice fed for 12 weeks. (D) The rate of CD44^high^CD62L^+^/CD4^+^ T cells in mice fed for 4 weeks. (E) The rate of CD44^high^CD62L^+^/CD4^+^ T cells in mice fed for 12 weeks. (F) The rate of CD44^low^CD62L^+^/CD4^+^ T cells in mice fed for 4 weeks. (G) The rate of CD44^low^CD62L^+^/CD4^+^ T cells in mice fed for 12 weeks. (H) The rate of CD44^low^CD62L^-^/CD4^+^ T cells in mice fed for 4 weeks. (I) The rate of CD44^low^CD62L^-^/CD4^+^ T cells in mice fed for 12 weeks. Diet containing 5% miso was fed to C57BL/6 mice for 4 (n = 12) or 12 (n = 3) weeks. Spleen samples were collected and were analyzed using flow cytometry. Bars indicate mean ± S.D. P-values relative to the control using a t-test.
(TIF)

S4 Fig Comparison of intestinal microflora analyzed from feces of mice fed control or 5% miso diets. (A) β-diversity (weighted UniFrac distance). (B, C, D, E) Four α-diversity indexes, (B) PD whole tree, (C) Observed OTUs, (D) Shannon index, (E) Simpson index. (F) Overall composition of microbiota at the phylum level.
(TIF)

S5 Fig Comparison of gene expression levels in spleen cells treated with the miso or other ingredients. Heat maps of representative gene expression of (A) transcription factors, (B) cytokines and (C) CD molecules.
(TIF)

S6 Fig Injection apparatus. This device together with a cover glass is used to hold the target tissue by compression with a pump. The solution can be injected though a tube from an injection syringe.
(TIF)

S1 Video Intravital Ca^2+^ signaling by PBS or NaCl in the IECs of a mouse expressing YC3.60. A total of 0.1 ml of PBS was added at the time point of 1 min and a total of 0.1 ml of 1% NaCl in water was added at the time point of 2 min 30 s. A rainbow parameter indicates relative Ca^2+^ concentration.
(MP4)

S2 Video Intravital Ca^2+^ signaling by miso in the IECs of a mouse expressing YC3.60. A total of 0.1 ml of 10% miso in PBS was added at the time point of 1 min was added at the time point of 2 min 30 s. A rainbow parameter indicates relative Ca^2+^ concentration.
(MP4)
