# Supplementary figures and images for: Ingestion of miso regulates immunological robustness in mice
Source: PLoS One. 2022 Jan 21;17(1):e0261680. doi: 10.1371/journal.pone.0261680 (PMC8782471; doi:10.1371/journal.pone.0261680)

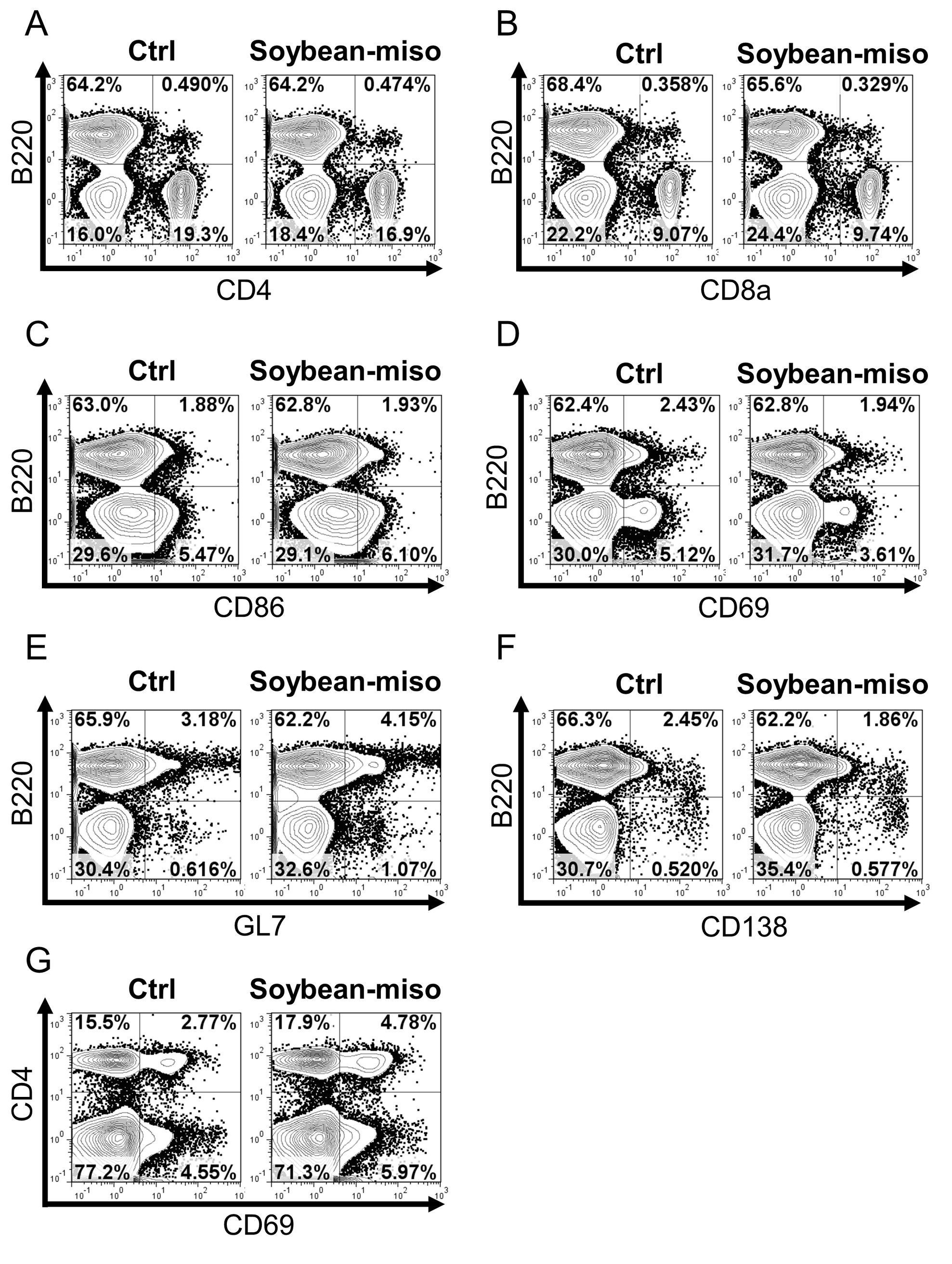

Supplement: S1 Fig — (A) B220 and CD4. (B) B220 and CD8a. (C) B220 and CD86. (D) B220 and CD69. (E) B220 and GL7. (F) B220 and CD138. (G) CD4 and CD69. (TIF) [file pone.0261680.s001.tif]

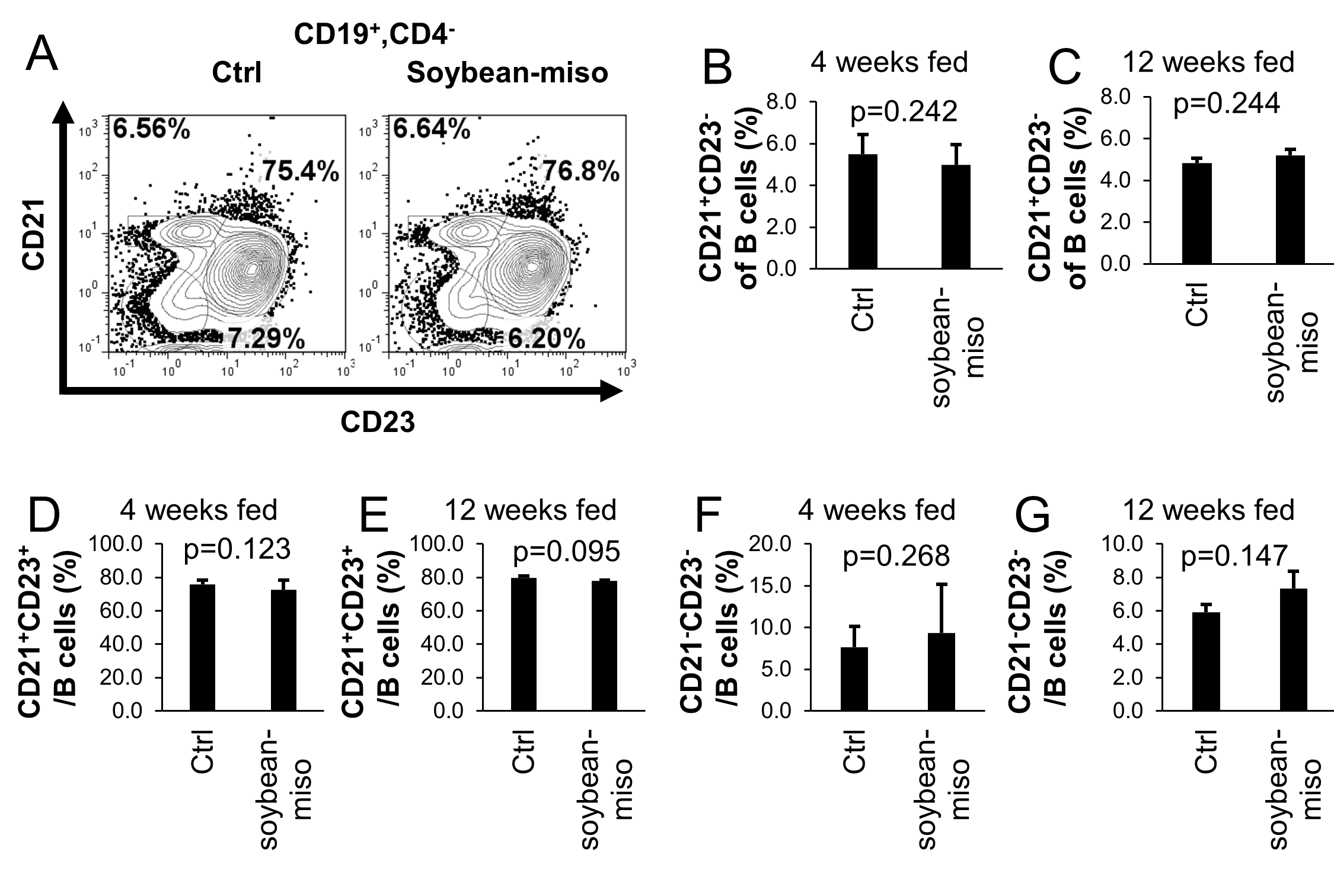

Supplement: S2 Fig — (A) Examples of the measurement by flow cytometryare shown. (B) The rate of CD21+CD23−/B cells in mice fed for 4 weeks. (C) The rate of CD21+CD23−/B cells in mice fed for 12 weeks. (D) The rate of CD21+CD23+/B cells in mice fed for 4 weeks. (E) The rate of CD21+CD23+/B cells in mice fed for 12 weeks. (F) The rate of CD21-CD23-/B cells in mice fed for 4 weeks. (G) The rate of CD21-CD23-/B cells in mice fed for 12 weeks. Diet containing 5% soybean-miso was fed to C57BL/6 mice for 4 (n = 12) or 12 (n = 3) weeks. Spleen samples were collected and were analyzed using flow cytometry. Bars indicate mean ± S.D. P-values relative to the control using a t-test. (TIF) [file pone.0261680.s002.tif]

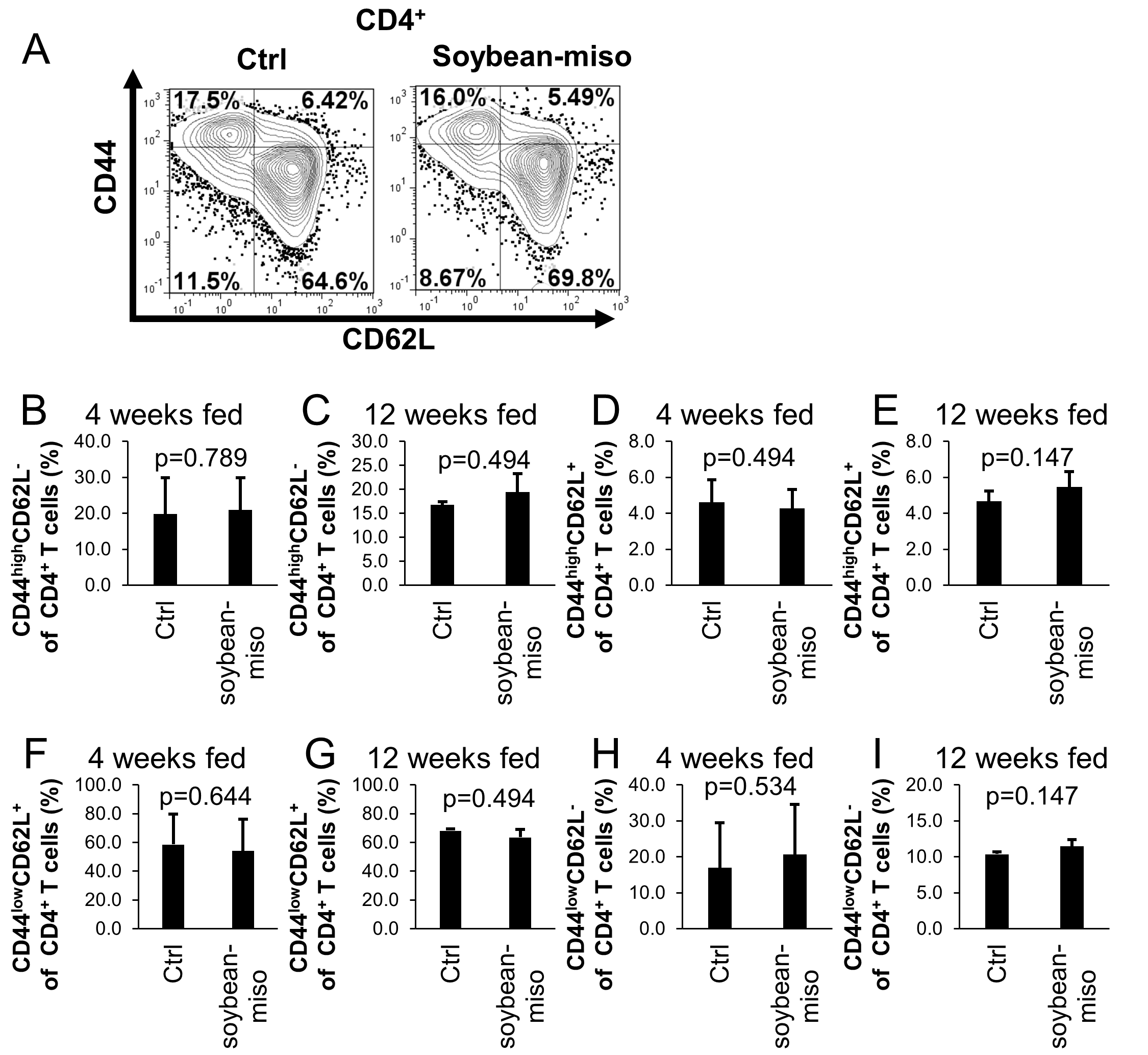

Supplement: S3 Fig — (A) Examples of the measurement by flow cytometry. (B) The rate of CD44highCD62L−/CD4+ T cells in mice fed for 4 weeks. (C) The rate of CD44highCD62L−/CD4+ T cells in mice fed for 12 weeks. (D) The rate of CD44highCD62L+/CD4+ T cells in mice fed for 4 weeks. (E) The rate of CD44highCD62L+/CD4+ T cells in mice fed for 12 weeks. (F) The rate of CD44lowCD62L+/CD4+ T cells in mice fed for 4 weeks. (G) The rate of CD44lowCD62L+/CD4+ T cells in mice fed for 12 weeks. (H) The rate of CD44lowCD62L-/CD4+ T cells in mice fed for 4 weeks. (I) The rate of CD44lowCD62L-/CD4+ T cells in mice fed for 12 weeks. Diet containing 5% miso was fed to C57BL/6 mice for 4 (n = 12) or 12 (n = 3) weeks. Spleen samples were collected and were analyzed using flow cytometry. Bars indicate mean ± S.D. P-values relative to the control using a t-test. (TIF) [file pone.0261680.s003.tif]

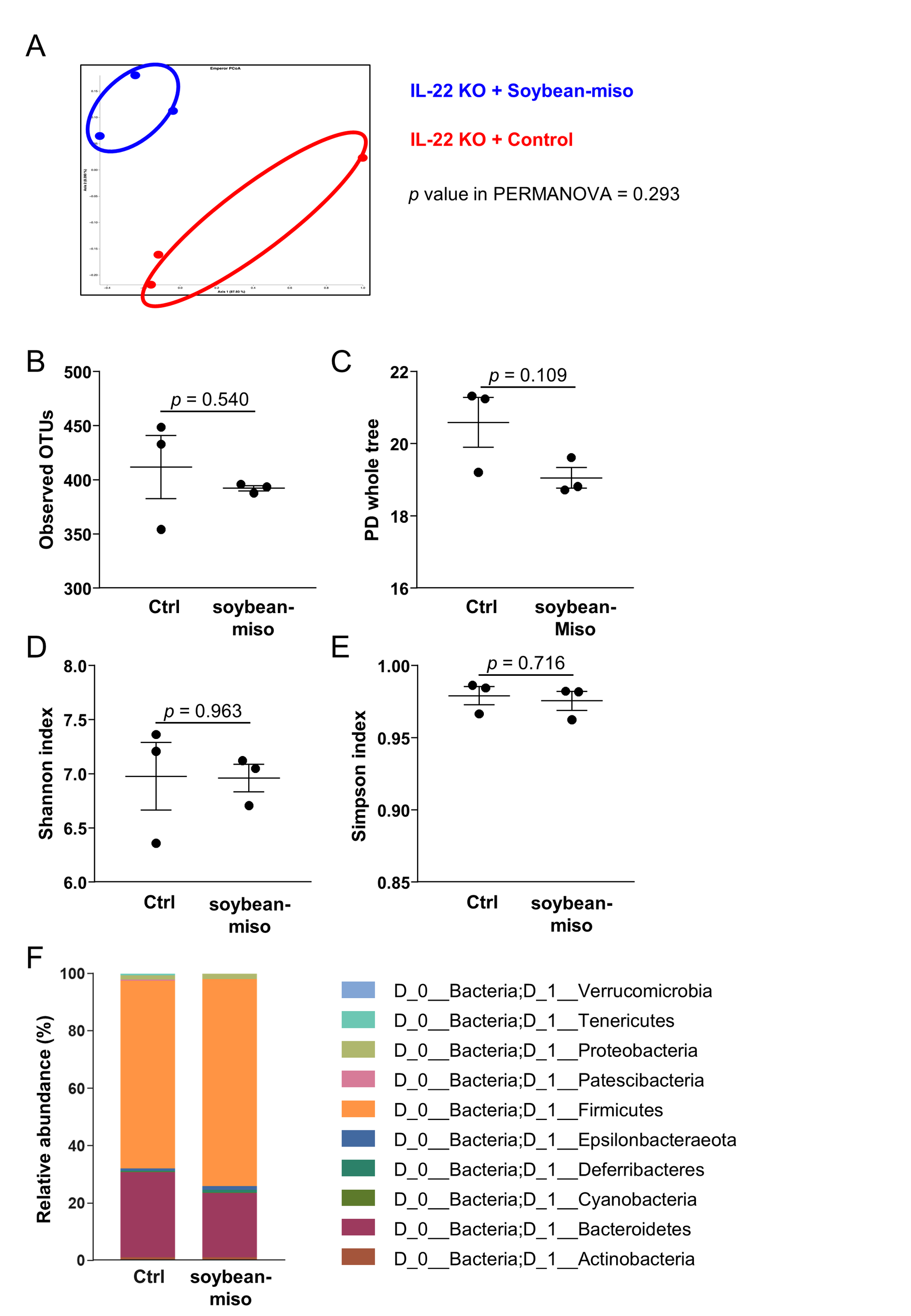

Supplement: S4 Fig — (A) β-diversity (weighted UniFrac distance). (B, C, D, E) Four α-diversity indexes, (B) PD whole tree, (C) Observed OTUs, (D) Shannon index, (E) Simpson index. (F) Overall composition of microbiota at the phylum level. (TIF) [file pone.0261680.s004.tif]

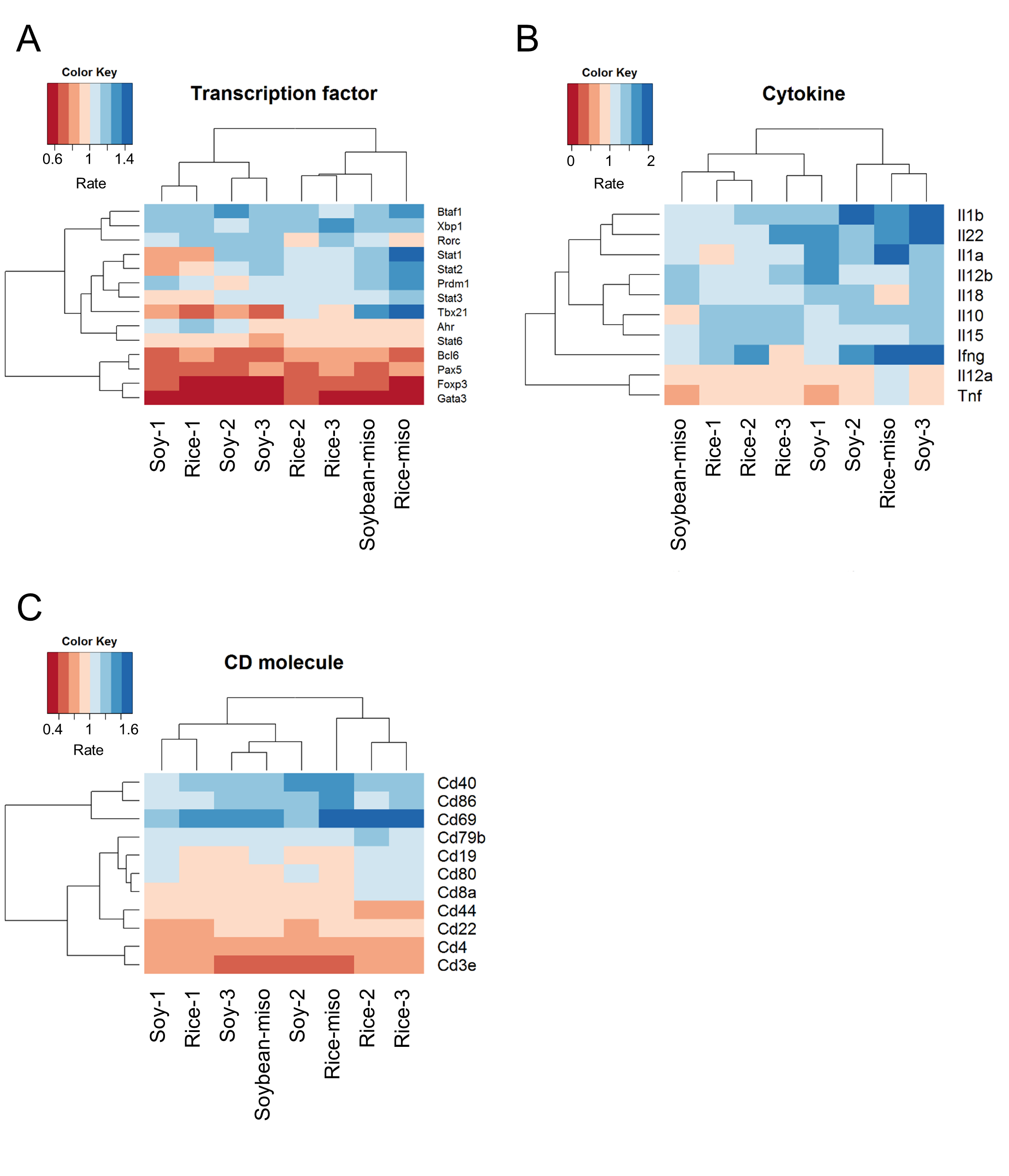

Supplement: S5 Fig — Heat maps of representative gene expression of (A) transcription factors, (B) cytokines and (C) CD molecules. (TIF) [file pone.0261680.s005.tif]

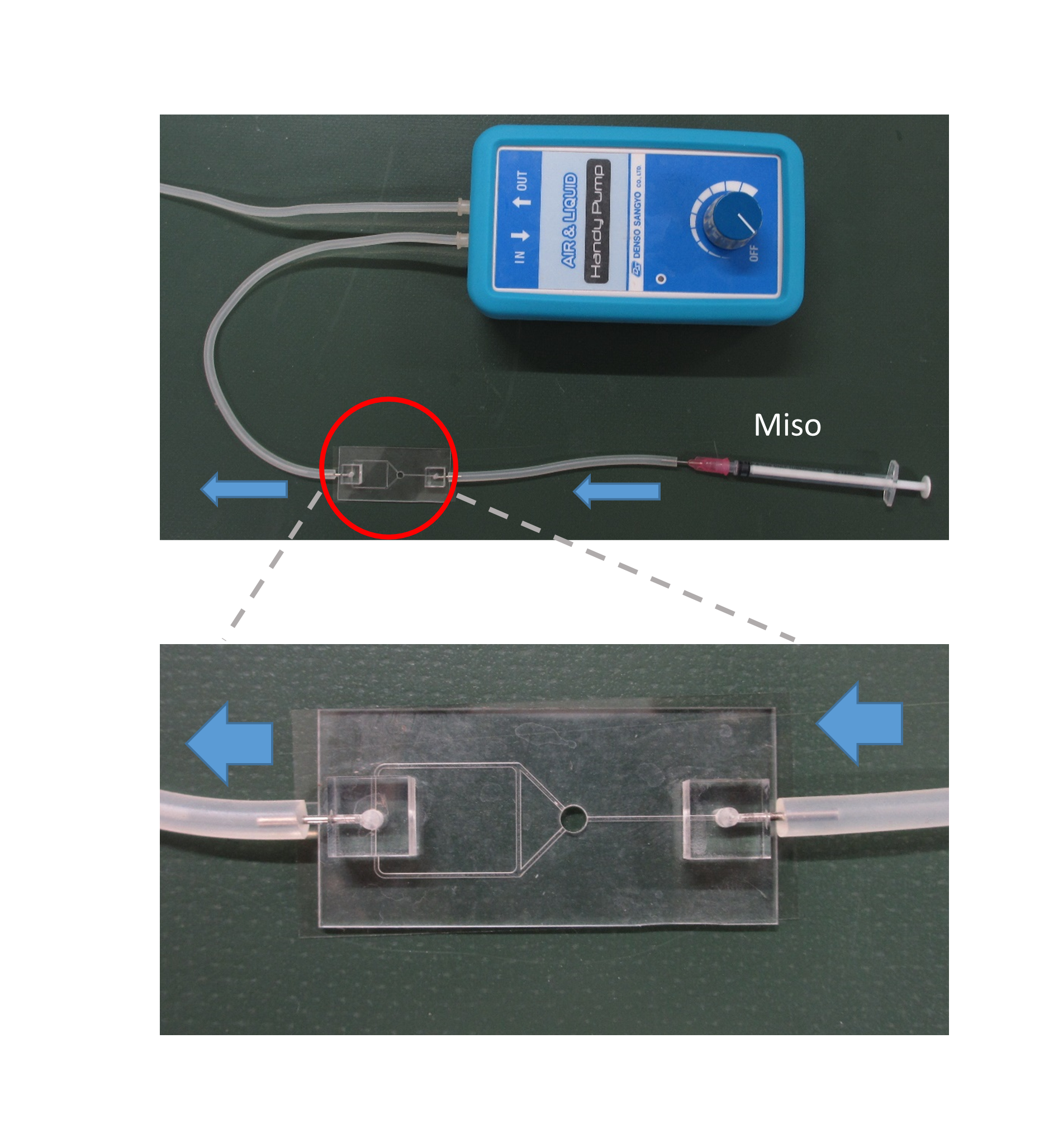

Supplement: S6 Fig — This device together with a cover glass is used to hold the target tissue by compression with a pump. The solution can be injected though a tube from an injection syringe. (TIF) [file pone.0261680.s006.tif]
